# Promoter hypermethylation of early B cell factor 1 (EBF1) is associated with cholangiocarcinoma progression

**DOI:** 10.7150/jca.52378

**Published:** 2021-03-05

**Authors:** Napat Armartmuntree, Apinya Jusakul, Chadamas Sakonsinsiri, Watcharin Loilome, Somchai Pinlaor, Piti Ungarreevittaya, Chern Han Yong, Anchalee Techasen, Kanokwan Imtawil, Ratthaphol Kraiklang, Nattawan Suwannakul, Waleeporn Kaewlert, Timpika Chaiprasert, Raynoo Thanan, Mariko Murata

**Affiliations:** 1 Department of Biochemistry, Faculty of Medicine, Khon Kaen University, Khon Kaen 40002, Thailand.; 2 Cholangiocarcinoma Research Institute, Khon Kaen University, Khon Kaen 40002, Thailand.; 3 Faculty of Associated Medical Sciences, Khon Kaen University, Khon Kaen 40002, Thailand.; 4 Department of Parasitology, Faculty of Medicine, Khon Kaen University, Khon Kaen 40002, Thailand.; 5 Department of Pathology, Faculty of Medicine, Khon Kaen University, Khon Kaen 40002, Thailand.; 6 Laboratory of Cancer Epigenome, Division of Medical Science, National Cancer Center Singapore, Singapore.; 7 Faculty of Public Health, Khon Kaen University, Khon Kaen 40002, Thailand.; 8 Department of Environmental and Molecular Medicine, Mie University Graduate School of Medicine, Mie 514-8507, Japan.

**Keywords:** cholangiocarcinoma, DNA methylation, EBF1, tumor progression, epigenetics

## Abstract

DNA hypermethylation in a promoter region causes gene silencing via epigenetic changes. We have previously reported that early B cell factor 1 (EBF1) was down-regulated in cholangiocarcinoma (CCA) tissues and related to tumor progression. Thus, we hypothesized that the DNA hypermethylation of EBF1 promoter would suppress EBF1 expression in CCA and induce its progression. In this study, the DNA methylation status of EBF1 and mRNA expression levels were analyzed in CCA and normal bile duct (NBD) tissues using a publicly available database of genome-wide association data. The results showed that the DNA methylation of EBF1 promoter region was significantly increased in CCA tissues compared with those of NBD. The degree of methylation was negatively correlated with EBF1 mRNA expression levels. Using methylation-specific PCR technique, the DNA methylation rates of EBF1 promoter region were investigated in CCA tissues (n=72). CCA patients with high methylation rates of EBF1 promoter region in the tumor tissues (54/72) had a poor prognosis. Higher methylation rates of EBF1 promoter region have shown in all CCA cell lines than that of an immortal cholangiocyte cell line (MMNK1). Upon treatment with the DNA methyltransferase inhibitor 5-Aza-dC, increased EBF1 expression levels and reduced DNA methylation rates were observed in CCA cells. Moreover, restoration of EBF1 expression in CCA cells led to inhibition of cell growth, migration and invasion. In addition, RNA sequencing analysis suggested that EBF1 is involved in suppression of numerous pathways in cancer. Taken together, DNA hypermethylation in the EBF1 promoter region suppresses EBF1 expression and induces CCA progression with aggressive clinical outcomes.

## Introduction

Epigenetics is a process that affects gene activity and expression without any changes in DNA sequences or genotype. Epigenetic processes include DNA methylation, histone modification and RNA-directed DNA methylation 1, 2. DNA methylation and histone modification are mostly observed in all organisms, whereas RNA-directed DNA methylation is found in plants. Histone modification and DNA methylation exert synergetic epigenetic regulatory effects 3. Therefore, DNA methylation is an epigenetic marker and plays a key role in carcinogenesis 4. This process is catalyzed by DNA methyltransferases (DNMTs), which transfer a methyl group from S-adenosylmethionine to a cytosine nucleotide molecule in regions known as CpG sites or CpG islands 5. In general, DNA hypermethylation of CpG islands which are located in a promoter region of gene causes silencing of the corresponding gene, whereas DNA hypomethylation at promoter regions promotes gene expression. DNA hypermethylation of promoter regions of many tumor suppressor genes suppresses their related gene expression and induces carcinogenesis 6.

Early B cell factor 1 (EBF1) is a transcriptional factor playing important roles in the differentiation of multiple stem cell lineages into mature cells 7-9. EBF1 is a tumor suppressor in leukemia 10, breast cancer 11 and cholangiocarcinoma (CCA) 12. EBF1 suppression is mediated via different mechanisms such as genomic loss in breast cancer 11, point mutation in pancreatic ductal adenocarcinoma 13, overexpression of EBF1 inhibitors (ZNF423 and ZNF521) in many cancers, such as CCA 14, acute lymphoblastic leukemia 15 and nasopharyngeal carcinoma 16, and aberrant expression of microRNAs targeting EBF1 in white adipose tissues 17. However, little is known about the importance of epigenetic changes in suppression of EBF1 expression.

CCA, a cancer arising from biliary epithelial cells, is the most common form of cancer in the Northeast region of Thailand including Khon Kaen Province where *Opisthorchis viverrini* (*O. viverrini*) infection is highly endemic 18. This small liver fluke has been classified as a major cause of CCA (group 1 carcinogen) by the International Agency for Research on Cancer 19. Several studies have strongly supported that oxidative stress was induced by *O. viverrini*-mediated inflammation. Oxidative stress is a major CCA driver and associated with biological damages, genetic instability, bile duct cell proliferation and pathological change in bile duct epithelium 19-22. When cholangiocyte cell lines were treated with hydrogen peroxide for a long-term, they increased cancer cell properties such as high expression levels of antioxidant enzymes (e.g., catalase (CAT), superoxide dismutase-2 (SOD2)) and high cell proliferation rates compared to the parental cells. Moreover, oxidative stress induces epigenetic changes through induction of the expression of DNMT1 23. The downregulation of EBF1 expression has recently been investigated in CCA patient tissues and it induced tumorigenic and stem cell properties of the cholangiocyte cells (MMNK1) 12. According to the previous findings, 47% of CCA tissues had EBF1 downregulation and increased oxidative stress marker. Furthermore, chronic exposure of cholangiocyte cells to oxidative stress could inhibit EBF1 expression, suggesting that prolonged oxidative stress could inhibit EBF1 expression 12. However, it should be noted that 53% of EBF1 downregulation in CCA tissues were not related to oxidative stress 12. In the present study, we hypothesized that the downregulation of EBF1 expression was not only resulted from oxidative stress, but also from the DNA hypermethylation of the EBF1 promoter of CCA cells, that may lead to CCA progression with aggressive clinical outcomes.

Here, we aimed to analyze the DNA methylation patterns and mRNA expression levels of EBF1 gene using the online data downloaded from the NCBI Gene Expression Omnibus (GEO) database (accession number GSE89803) 24. The significance of DNA methylation status of EBF1 promoter region of CCA tissues, CCA cell lines, and an immortalized cholangiocyte cell line (MMNK1) was confirmed using methylation-specific polymerase chain reaction (MSP) analysis. Associations between DNA methylation status and clinicopathological data including age, sex, tumor stage, histological types, metastasis status and survival days after surgery were evaluated. Moreover, CCA cell lines and MMNK1 cell line were treated with the DNMT inhibitor 5-Aza-dC and subjected to MSP and EBF1 expression analyses using real-time quantitative polymerase chain reaction (RT-qPCR) and immunocytochemistry **(**ICC**)** techniques to confirm that the EBF1 expression level of CCA is regulated by its promoter hypermethylation. To evaluate that the increase of EBF1 expression can be used for CCA targeted therapy, the functional roles of EBF1 related to tumor suppression were investigated further in EBF1-overexpressing CCA cells.

## Materials and Methods

### Human CCA tissues

All frozen tissues and paraffin-embedded tissues from the CCA patients were obtained from the specimen bank of the Cholangiocarcinoma Research Institute, Khon Kaen University, Thailand. CCA tissues and normal bile ducts from tumor adjacent areas were collected from the patients that were admitted at the surgical wards of Srinagarind Hospital, Khon Kaen University. The study was approved by the Ethic Committee for Human Research, Khon Kaen University, Thailand (HE571283 and HE611577).

### Illumina 450K data methylation analysis

**Figure 1:**
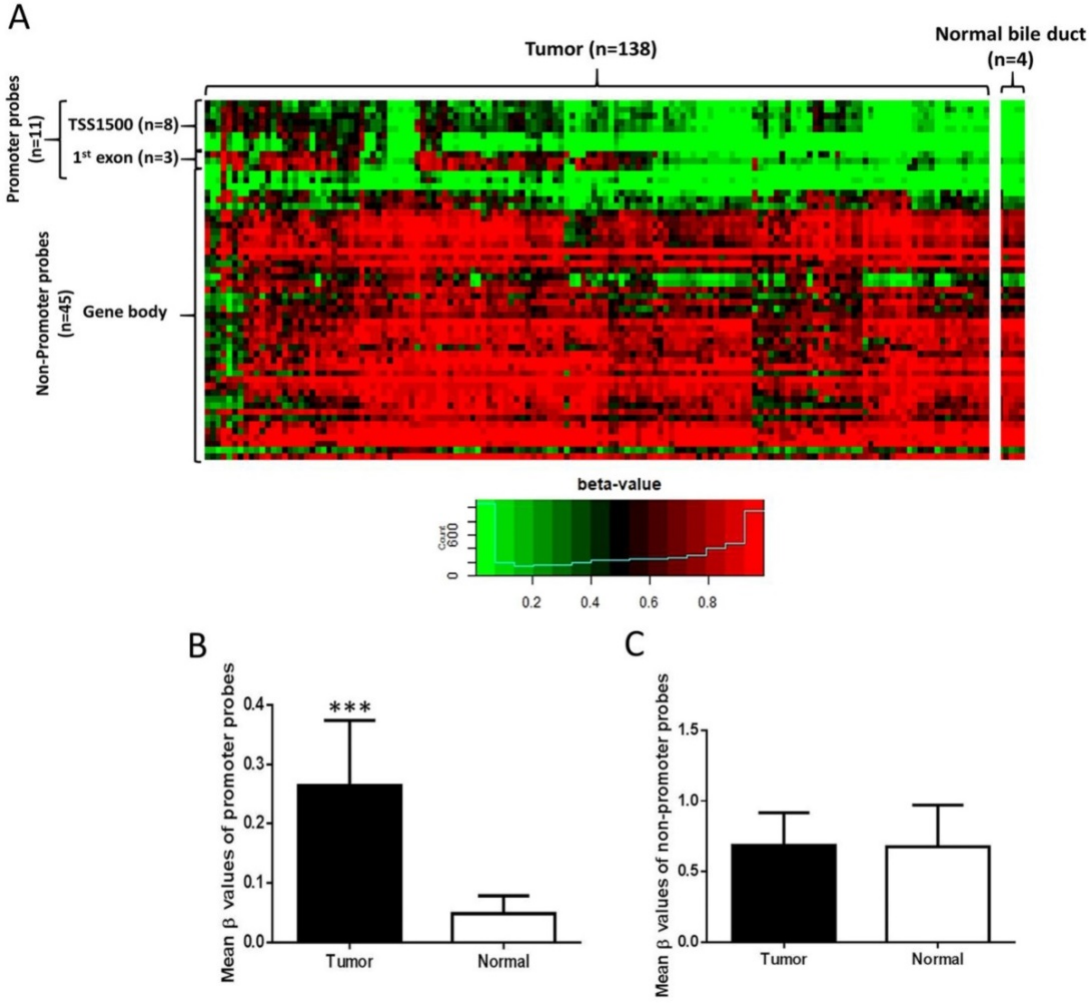
Distribution of EBF1 methylation in CCA. (A) Heat map of all EBF1 methylated probes. Hierarchical clustering was performed using all methylated EBF1 probes. TSS1500 covers -200 to -1500 nt upstream of translation start site (TSS). Box plots illustrated the methylation β-values between normal bile duct (Normal) and CCA tissue (Tumor) of the EBF1 promoter region (B) and non-promoter region (C). Statistically significant differences (***P < 0.0001) in group β-values by two-sided t-test.

Illumina Infinium Human Methylation 450K Methylation data (138 CCA tissues and 4 normal bile duct samples) were downloaded from the NCBI GEO database (accession number GSE89803). Data were subjected to pre-processing using the “minfi” 25 and “wateRmelon” 26 R packages. Methylation levels of CpG sites were calculated as β-values. β-value is a continuous variable between 0 and 1 and a value of 0 corresponds to no methylation, while a value of 1 corresponds to 100% methylation at the specific CpG site analyzed. Fifty-six probes proximal to EBF1 were tested for methylation levels between normal bile duct and CCA samples (Figure 1A). Annotations of CpG probes to genomic features were obtained from the “Illumina Human Methylation 450K manifest” package 25, 26. Probes annotated to the transcription start site (TSS) 1500 or first exon of EBF1 were considered the promoter probes, while the remainder (annotated to gene body and 3' UTR) were considered non-promoter probes, giving 11 promoter probes and 45 non-promoter probes. A probe was considered hypermethylated if the difference in mean beta values between the two groups was at least 0.2, and FDR < 0.05 (two-sided t test, with Benjamini-Hochberg correction for multiple hypothesis testing).

### DNA extraction

Genomic DNA was extracted from frozen human CCA tissues (n = 72) and cell pellets, as previously described with some modifications 27. Frozen tissues (50 mg) or cell pellets (10^6^ cells) were homogenized in a lysis solution (1% [w/v] Triton X-100, 0.32 M saccharose, 5 mM magnesium chloride [MgCl_2_], and 10 mM Tris-HCl pH 7.5) and centrifuged at 10,000 ×g for 5 min. The pellets were collected and repeatedly washed, followed by incubation with 8 µL of RNase A (1 mg/mL) and 200 µL of enzyme reaction solution (1% [w/v] sodium dodecyl sulfate [SDS], 5 mM ethylenediaminetetraacetic acid [EDTA], and 10 mM Tris-HCl, pH 8.0) for 20 min. The samples were treated with 10 µL of proteinase K (17 mg/mL) at 37 ºC for 3 h with occasional mixing. After incubation, 300 µL of 7.6 M sodium iodide (NaI) solution was added to the mixture, followed by the addition of 500 µL of absolute isopropanol. The mixture was centrifuged at 10,000 ×g for 10 min and the extracted DNA pellet was washed with 40% isopropanol. The pellet was washed twice with 70% ethanol and re-suspended in Tris-EDTA buffer (pH 8.0). The DNA solution was stored at -20 °C until analysis. DNA concentration and purity were determined with NanoVue™ spectrophotometer (GE Healthcare, NJ, USA).

### Bisulfite conversion of genomic DNA

Genomic DNA was subjected to bisulfite conversion using EZ DNA Methylation-Gold™ Kit (Zymo Research, CA, USA) according to the manufacturer's instructions. In brief, 130 µL of CT conversion reagent was added to 1 μg of DNA in a total volume of 20 µL in a 200-µL microtube. Bisulfite conversion was performed as follows: incubation of samples at 98 °C for 10 min, followed by 64 °C for 2.5 h. The bisulfite-treated DNA was washed and eluted as per the manufacturer's protocol. The treated samples were stored at -20 °C until analysis. Bisulfite-treated DNA samples were subjected to PCR using calponin-specific primers for the confirmation of their complete bisulfite-mediated conversion 28.

### Methylation-specific polymerase chain reaction (MSP) analysis

EBF1 methylation rates were determined using a MSP assay. Bisulfite-treated DNA samples were used as templates. The methylation primers (M) and unmethylation primers (U) specific for amplification of EBF1 promoter region are shown in Table S1. Both primers were designed using The BiSearch web server 29, 30. The PCR reaction condition is shown in Table S2. Primer sets were designed to bind at the same region, and the PCR products were 142 bp (Chr5:159100738-159100879, -821 to -963 bp upstream of EBF1 transcript start site) in length. PCR reactions were performed on C-1000 Thermal cycle (Bio-RAD, CA, USA) and the products were separated on 1.5% agarose gel, stained with ethidium bromide, and visualized under UV illumination (Bio-Rad, CA, USA). The products (142 bp) were subjected to DNA sequencing (Pacific Science Company, WA, USA). The 142 bp PCR products showed a 100% match with the designed bisulfite-treated DNA sequences (methylated and unmethylated products; Supplementary Figure S1). Methylation patterns of KKU-213B and MMNK1 were used as inter-batch controls. The quantification of 142 bp band intensity was performed with ImageJ software version 1.47. Methylation rate (M/[M + U]) was calculated from the intensities of PCR products of M and U primers in each case.

### Cell lines

Human CCA cell lines, including KKU-213A, KKU-213B and KKU-213C were obtained from the Cholangiocarcinoma Research Institute, Khon Kaen University, Thailand. An immortalized cholangiocyte cell line (MMNK1) was obtained from Okayama University, Japan 31. KKU-213A, KKU-213B and KKU-213C have been recently characterized and renamed from KKU-213, KKU-214 and KKU-156, respectively 32. All cell lines were cultured in Ham's F12 medium (Invitrogen, MA, USA) supplemented with 10% fetal calf serum, 100 U/mL of penicillin, and 100 µg/mL of streptomycin (complete media) at 37 °C in a humidified incubator maintained in an atmosphere of 5% CO_2_. Cells were subcultured once they reached confluency and media were changed once every 2 to 3 days.

### Treatment of cells with 5‑aza‑2′‑deoxycytidine (5-Aza-dC)

The 5-Aza-dC (Sigma-Aldrich, MO, USA) was dissolved in 100% dimethyl sulfoxide (DMSO) to obtain a 200 mM stock solution, aliquoted and stored at -20 °C until analysis. CCA and MMNK1 cell lines were seeded (2 × 10^5^ cells/mL) in 100 × 20 mm cell culture dishes and continuously treated with 1 µM of 5-Aza-dC containing 0.05% DMSO in complete medium (10 mL) for 6-12 days. The cells were harvested for MSP, RT-qPCR and ICC techniques. In addition, cells treated with 0.05% DMSO in complete medium without 5-Aza-dC were used as controls.

### Transient overexpression of EBF1

The EBF1 expression vector (EBF1 vector) and control vector (empty vector) were synthesized (Dharmacon Inc., Lafayette, CO, USA) and extracted from *Escherichia coli* (*E. coli*) cells containing a EBF1 vector (pLOC-TurboRFP containing EBF1 gene) or a control vector (pLOC-TurboRFP without EBF1 gene). Plasmid DNA extraction from *E. coli* was performed using GF-1 Plasmid DNA Extraction Kit (Vivantis Technologies, Selangor, Malaysia) following the manufacturer's instructions. KKU-213A cell line was maintained in complete media at 37 °C in a humidified incubator with 5% CO_2_. Cells were seeded in six-well plates (1.5 × 10^5^ cells/well) and 70-80% confluent cells were transiently transfected with 1 µg of control vector or EBF1 vector using Lipofectamine 2000 (Invitrogen, MA, USA) according to the manufacturer's recommendations. Twenty-four hours after transfection, cells were collected for various experiments. Cells transfected with control vector served as control cells.

### Detection of mRNA levels by RT-qPCR

Trizol^®^ reagent (Invitrogen, MA, USA) was used to extract total RNA from cell pellets as per the manufacturer's protocol. For cDNA synthesis, 2 µg of total RNA was converted to cDNA using High-Capacity cDNA Reverse Transcription Kit (Applied Biosystems, CA, USA). EBF1, β-actin, IL-6, COX-2, MMP-9, Oct3/4, CAT, SOD2 mRNA expression levels were analyzed with TaqMan gene expression assay using TaqMan probes (EBF1, Hs00395513_m1; β-actin, Hs99999903_m1; IL-6, Hs00174131_m1; COX-2, Hs00153133_m1; MMP-9, Hs00957562_m1; Oct3/4, Hs04260367_gH; CAT, Hs00156308_m1 and SOD2, Hs01553554_m1) on ABI-7500 RT-qPCR system (Applied Biosystems, CA, USA). Relative mRNA expression (fold change) was analyzed with a cycle threshold (Ct) in a linear range of amplification with β-actin as an internal control.

### Immunofluorescence staining

CCA cells and transient CCA cells (50,000 cells/well) were seeded in eight-well chamber slide for overnight cultivation. The cells were fixed with 4% formaldehyde-containing phosphate-buffered saline (PBS) for 10 min at room temperature. Cells were incubated in 0.5% (v/v) Triton X-100 solution to change their membrane permeability, followed by incubation with 2N hydrochloric acid (HCl) (30 min) for antigen retrieval. Nonspecific binding was blocked using 1% (w/v) skim milk in PBS for 30 min. Cells were incubated with 5 µg/mL of rabbit anti-EBF1 polyclonal antibody (Abcam, MA, USA) at room temperature for overnight, followed by treatment with Alexa Fluor^®^ 594 goat anti-rabbit IgG (Invitrogen, MA, USA). Nucleus was staining with DAPI Fluoromount-G^®^ (Southern Biotech, Alabama, USA). The stained cells were analyzed using a fluorescent microscope (Olympus BX53F, Tokyo, Japan).

### Immunocytochemistry of cell lines

Cells (60,000 cells/well) were seeded in 48-well plates for overnight cultivation. The cells were fixed with 4% paraformaldehyde-containing phosphate-buffered saline (PBS) for 30 min at room temperature. Cells were incubated in 0.2% (v/v) Triton X-100 solution to change their membrane permeability, followed by incubation with PBS containing 0.3% (v/v) hydrogen peroxide (30 min). Nonspecific binding was blocked using 3% (w/v) bovine serum albumin (BSA) in PBS for 30 min. Cells were incubated with 3 µg/mL of rabbit anti-EBF1 polyclonal antibody (Abcam, MA, USA) at room temperature for overnight, followed by treatment with peroxidase-conjugated Envision^TM^ secondary antibody (DAKO, Glostrup, Denmark). The color was developed with 3,3'-diaminobenzidine tetrahydrochloride (DAB) substrate kit (Vector Laboratories, Inc., CA, USA) and washed with distilled water. The stained cells were dehydrated with ethanol and air dried for overnight. The stained cells were analyzed using an inverted microscope. The semi-quantitative analysis was calculated in ImageJ Fiji software using the method described by Crowe and Yue 33.

### Wound healing assay

Cells were seeded in a 24-well plate and cultured in complete medium at 37 °C in a humidified incubator maintained in an atmosphere of 5% CO_2_. After reaching ~90% confluence, cell monolayers were scratched using a 200-µL pipette tip and rinsed thrice with PBS to remove cell debris. Cell migration in the wound area was observed under a phase-contrast microscope and digitally photographed. Wound healing was measured using images and the migration area was calculated from the results of the area of original wound minus the area of wound during healing divided by the area of original wound.

### Cell invasion assay

The invasive ability of cells was assessed using Boyden chamber with an insert filter coated with Matrigel Matrix coating solution (8-µm pore size; Corning, NY, USA). The assay kit was reconstituted by adding serum-free medium into the upper chamber and complete medium into the lower chamber for approximately 1 h. Before seeding the cells, serum-free medium in the upper chamber was removed and replaced with cells (3 × 10^4^ cells) in serum-free medium. After 24 h incubation, the non-invading cells in the upper chamber were removed with a cotton swab. The invaded cells attached under the filter were fixed with 100% methanol for 30 min and stained with hematoxylin for overnight. The quantification of invading cells was performed by counting six random fields at ×200 magnification under a light microscope.

### Hydrogen peroxide (H_2_O_2_) treatment and Sulforhodamine B (SRB) assay

For H_2_O_2_ exposure, cells (3 × 10^3^ cells/well in 100 µL volume) were plated into 96-well plates in triplicate, after 12 h of cell adhesion, the cells were exposed to different concentrantion of H_2_O_2_ (Sigma-Aldrich, MO, USA) for 72 h. The cells were then fixed with 10% (w/v) trichloroacetic acid (TCA) and subjected to cell density analysis using SRB colorimetric assay. The cells were dried at 60 °C for 30 min and treated with SRB (0.4% [w/v] in 1% [v/v] of acetic acid) and incubated for 1 h. Excess dye was removed by repeatedly washing with 1% (v/v) acetic acid. The protein-bound dye was dissolved in 10 mM of Tris-based solution (pH 10.5) for 1 h with gentle shaking. The optical density (OD) was spectrophotometrically determined at 540 nm wavelength using a microplate reader (Sunrise, Tecan Austria GmbH).

### RNA sequencing (RNA-seq) and data processing

NucleoSpin^®^ RNA kit was used to extract the total RNA from KKU-213A cells treated with empty vector (control cells; n=2) or EBF1 plasmid vector (EBF1-overexpressing cells; n=2). All obtained RNA samples were subjected to NovaSeq 6000 Sequencing System at Takara Company (Shiga, Japan). For data processing pipeline, Genedata Profiler Genome software** (**version 11.0.11**)** was used for base calling. STAR (version 2.5.3a) was used to align RNA sequencing reads to reference genome. The RNA sequencing of EBF1-overexpressing CCA cells were uploaded to the NCBI GEO database **(**accession number GSE162836) and provided the processed RNA sequencing data as Supplementary Table S3. A *P* value < 0.05 was deemed as a significant differential gene expression. Fold-change **(**FC**)** was calculated by FPKM values of test group divided by FPKM values of control group. The FC > 1 and FC < 1 are used as cutoffs for up-regulated and down-regulated genes, respectively. The lists of up-regulated and down-regulated genes were submitted to DAVID Bioinformatics Resources version 6.8 online server (https://david.ncifcrf.gov) and KEGG pathway enrichment analysis.

### Statistical analysis

Statistical analysis was performed using SPSS software version 17.0 (IBM Corporation, USA). The association of methylation status of EBF1 promoter with patient clinicopathological factors assessed by Fishers' exact test was analyzed. Survival analysis was performed using the Kaplan-Meier estimate with log-rank test. Levels of mRNA and protein expression were compared with the Student's t-test. The difference in the methylation levels between normal bile duct and CCA was analyzed using two-sided t-test and Benjamini-Hochberg correction for multiple hypothesis testing, with FDR < 0.05 considered statistically significant.

## Results

### The methylation status and mRNA expression levels of EBF1 in a publicly available genome-wide database

The methylation status of EBF1 in a publicly available genome-wide DNA methylation database (GSE89803) of CCA and micro-disectioned normal bile ducts tissues from the *O. viverrini* infection edemic (Northeast Thailand) and non-endemic areas (Singapore and Romania) was investigated 24. Methylation values of CpG sites proximal to EBF1 were compared between tumors (n = 138) and normal bile ducts (n = 4) (Figure 1A). Among 56 EBF1 probes, 11 of 11 probes located at EBF1 promoter region (TSS1500 and first exon) were significantly hypermethylated in CCA tumors compared with normal bile ducts (Figure 1B), and the methylation levels of 4 (located at TSS1500**)** of 11 probes were negatively correlated with the EBF1 mRNA expression levels as shown in Table S4. In contrast, the methylation levels of 45 non-promoter probes were not different between CCA and normal tissues (Figure 1C).

### Methylation status of EBF1 in human CCA tissues based on the clinicopathological data

**Figure 2:**
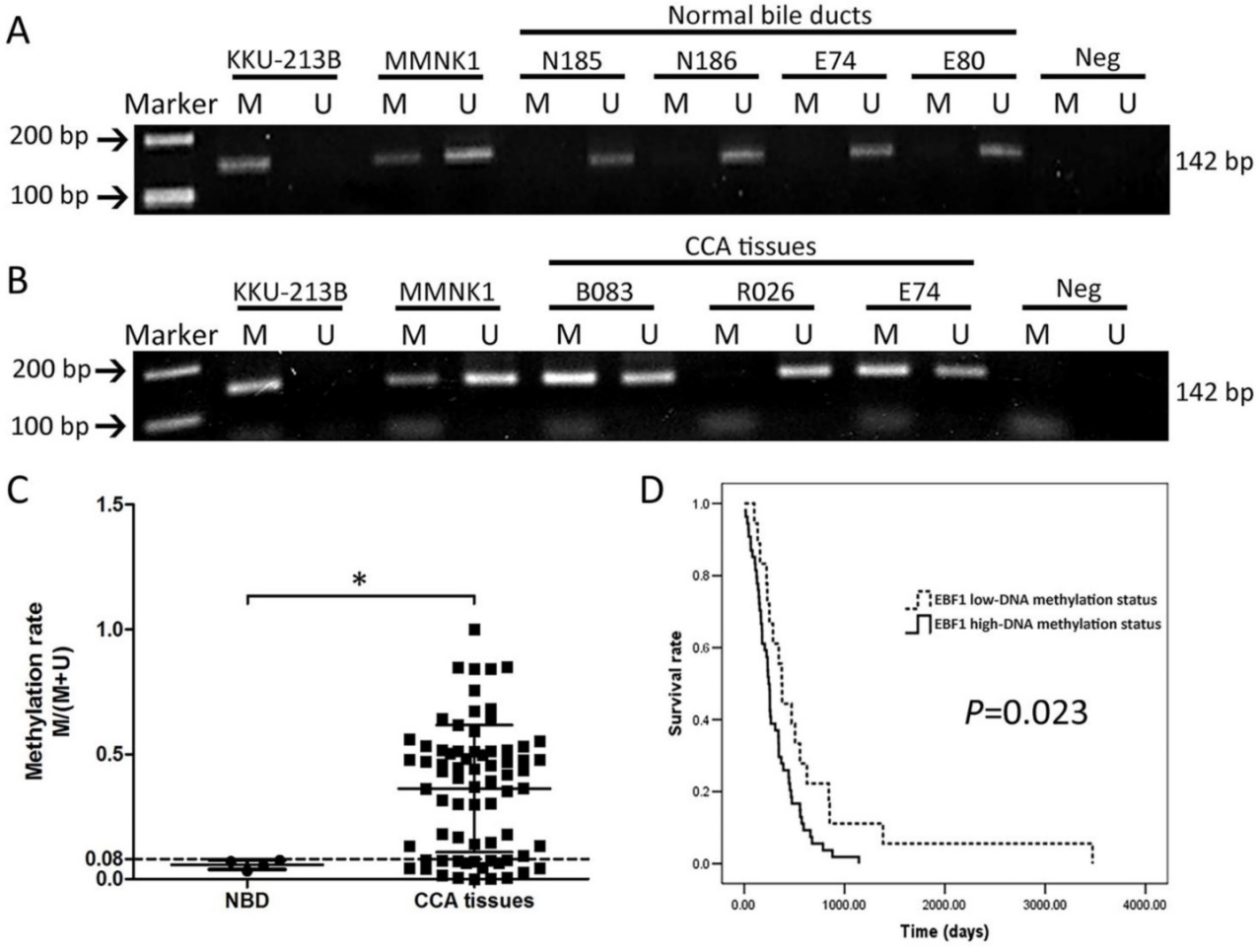
MSP analyses of EBF1 promoter region in normal bile ducts (NBD) (A) and CCA tumor tissues (B). M = PCR product from methylation primers, U = PCR product from unmethylation primers. Methylation patterns of KKU-213B and MMNK1 cells were used as the inter-batch quality controls. (C) Methylation rates (M/[M + U]) in NBD (n = 4) and CCA (n = 72) tissues. *P* value was calculated with Mann-Whitney U test (*P = 0.015). (D) Kaplan-Meier analyses of survival rates of patients with CCA with low-DNA methylation (methylation rate ≤ 0.08; dashed line) and high DNA methylation (methylation rate > 0.08; solid line) of EBF1 promoter regions. *P* value was analyzed with log-rank test.

To confirm the DNA methylation array of the EBF1 promoter, alteration in methylation status of EBF1 was studied using a MSP technique. The methylation rate (M/[M + U]) was calculated from the intensities of the PCR products using a specific methylation primer set (M) and an unmethylation primer set (U). The methylation rates ranged from 0 to 1. Figure 2A and 2B illustrates an example of EBF1 methylation patterns in KKU-213B cell line, MMNK1 cell line, normal bile duct tissues and CCA tissues. The DNA methylation patterns of KKU-213B and MMNK1 cell lines were used as inter-batch controls. All normal bile ducts (NBD) tissues were positive with U primers only and showed low EBF1 methylation rates at the promoter region (Figure 2A). Most of CCA tissues showed positive for both primer types (M and U primers) except for few cases (as shown in case R026, Figure 2B) with U primers positive only. Focus on the DNA methylation patterns of NBD and CCA tissues of the case number E74 in Figure 2A and 2B, the PCR product of M primers was increased in CCA tissue compared to the individual normal bile duct tissue. The DNA methylation rates were significantly higher in CCA tissues (average methylation rate = 0.36 ± 0.25) compared to the NBD (average methylation rate = 0.05 ± 0.01) as shown in Figure 2C.

Then, the quartile 1 (Q1) value (0.08) of the methylation rates of CCA tissues was used as a cut-off point for low- and high-DNA methylation status. The results of correlation analyses between methylation status of EBF1 promoter region and the gender, age, CCA histology types, and metastatic stages of CCA patients showed no significant associations (Table S5). However, when association between methylation status of EBF1 promoter and survival analysis was determined with the Kaplan-Meier method and log-rank test, the patients with CCA exhibiting high EBF1 methylation status is associated with poor prognosis (*P* = 0.023), as shown in Figure 2D. The overall median survival time was 256 days (95% confidence interval [CI], 221.3-300.7). CCA patients with high EBF1 methylation status showed shorter survival (median, 243 days; 95% CI, 219.3-266.7) than those with low EBF1 methylation status (median, 374 days; 95% CI, 305.4-442.6).

### Methylation status and expression of EBF1 are regulated by DNMTs in CCA and cholangiocyte cells

**Figure 3:**
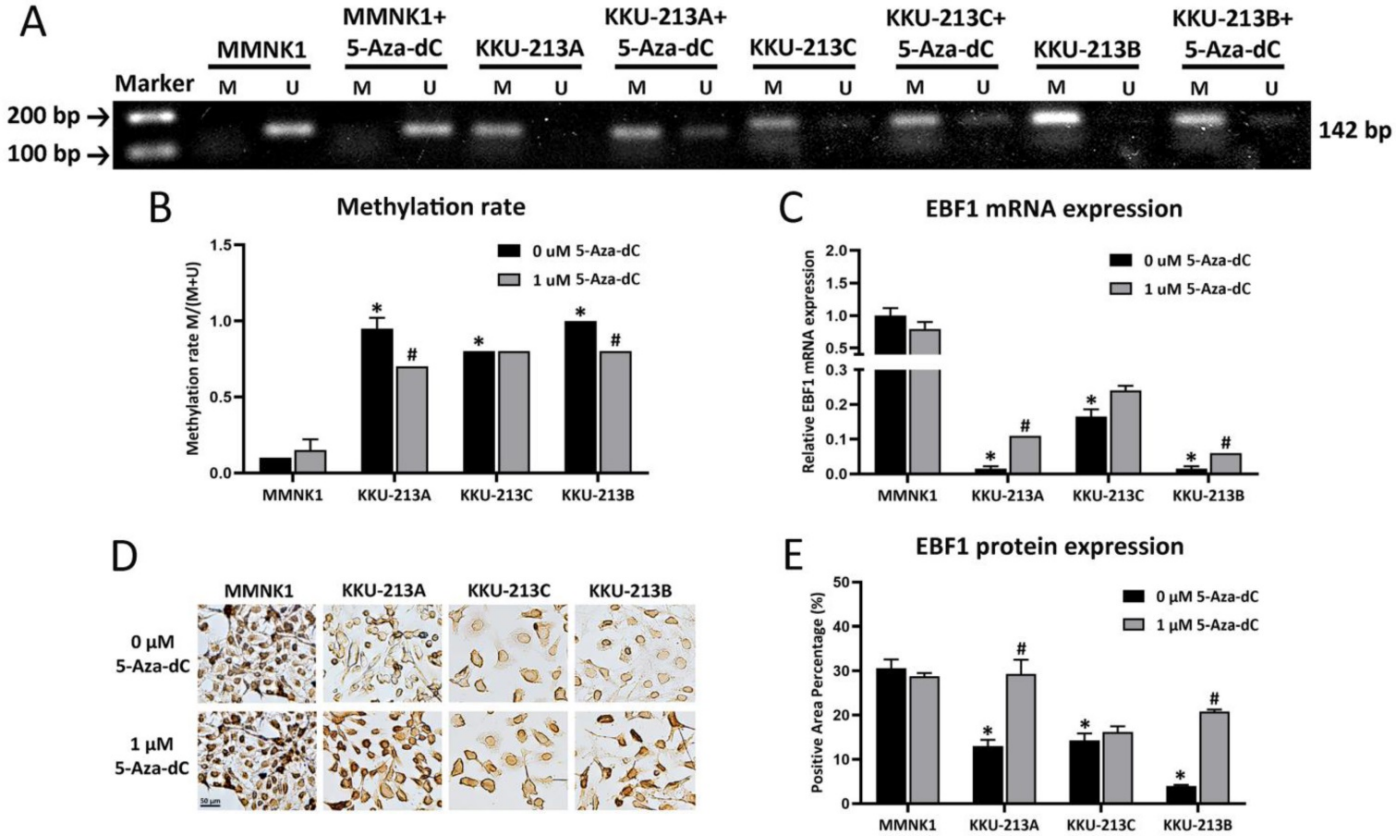
Effects of 5-Aza-dC treatment on DNA methylation status and expression of EBF1 (A) MSP analyses of EBF1 promoter region in an immortalized cholangiocyte (MMNK1) and CCA (KKU-213A, KKU-213C, KKU-213B) cell lines. M = PCR product from methylation primers, U = PCR product from unmethylation primers. (B) Graphical representation of the DNA methylation rates of MMNK1 and CCA cell lines. (C) mRNA expression levels of EBF1 in cells with or without 5-Aza-dC treatment for 6 days (KKU-213A) and 12 days (MMNK1, KKU-213C and KKU-213B). (D) Protein expression levels and (E) semi-quantification of EBF1 expression was detected by immunocytochemical staining. *P < 0.05 vs. MMNK1 cell; ^#^*P* < 0.05 vs. 5-Aza-dC untreated-CCA cells.

The methylation patterns of EBF1 promoter region of a cholangiocyte cell line (MMNK1) and CCA cell lines (KKU-213A, KKU-213C and KKU-213B) were examined. DNA methylation rates of the EBF1 promoter region of CCA cell lines (average methylation rate = KKU213A, 0.95 ± 0.007; KKU213C, 0.82 ± 0.001 and KKU213B, 0.97 ± 0.005) were significantly higher than that of the MMNK1 cell line (average methylation rate = 0.08 ± 0.04). To evaluate the importance of DNA methylation on EBF1 expression in the cholangiocyte and CCA cell lines, MMNK1 and CCA cells were treated with DNMT inhibitor, 5-Aza-dC, for 6 days. After 5-Aza-dC-treatment, CCA cell lines (KKU-213A and KKU-213B) showed significant reduction of the DNA methylation rates as shown in Figure 3A and Figure 3B. Notably, significant increases of EBF1 mRNA expression were observed in 5-Aza-dC treated-KKU-213A and KKU-213B cells (Figure 3C). Such singificant changes were not observed in 5-Aza-dC treated-MMNK1 and KKU-213C cells under the condition used. Immunocytochemical technique revealed protein levels were similar to gene expression levels, as shown in Figure 3D and Figure 3E. Higher concentration of 5-Aza-dC (5 µM) yieled significant increase of EBF1 mRNA expression in KKU-213C cells, but no change in MMNK1 cells (data not shown). These data suggest that induction of DNA methylation of EBF1 promoter results in EBF1 downregulation in CCA.

### Restored EBF1 expression suppresses migration, proliferation, and invasion of CCA cells

**Figure 4:**
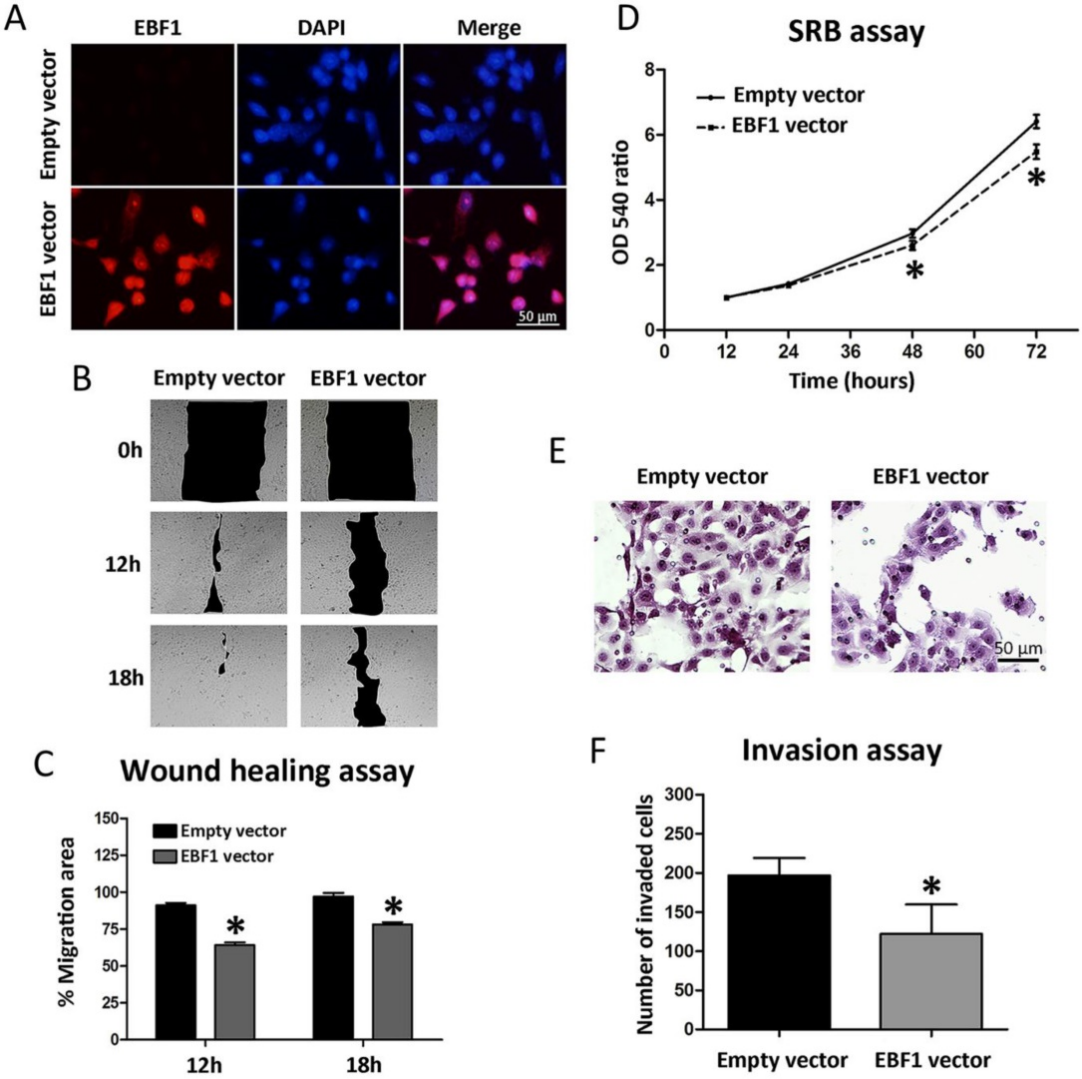
(A) EBF1 protein expression levels in KKU-213A cells transfected with EBF1 vector or with empty vector were measured using immunofluorescence staining. Original magnification is ×200 for all figures. (B) Wound healing assay under a microscope (×10), and (C) graphical data indicating the percentage of migration area determined by wound healing assay. (D) Effect of EBF1 overexpression on the proliferation of CCA cells was determined with SRB assay. Proliferation curve of KKU-213A cells transiently transfected with EBF1 vector (dashed line) as compared with empty vector (solid line). (E) Quantification of invading cells was performed by counting six random fields at ×200 under a light microscope. (F) Significant increase in the number of invasive cells in KKU-213A cells transfected with EBF1 vector as compared with control cells. *P* values are indicated by the asterisk (*) *P* < 0.05.

To see whether EBF1 overexpression can be used for CCA targeted therapy, we investigated further the roles of EBF1 in CCA cell progression. All CCA cell lines had low EBF1 expression 12. The functional roles of EBF1 in relation to CCA cell progression were studied in KKU-213A cells transfected with control or EBF1-overexpressing vectors. Figure 4A showed the efficiency of EBF1 overexpression in CCA cells using immunofluorescence techniques. After 24 h of transfection, the EBF1 protein expression levels were higher in the nucleus of EBF1-overexpressing CCA cells than in the control cells.

EBF1 overexpression also affected the lateral migration of CCA cells. An *in vitro* wound healing assay was performed with CCA cells transfected with EBF1 vector or control vector. Cell migration ability was monitored every 6 h until the gap closure. The results showed that the overexpression of EBF1 resulted in an apparent decrease in the migration activity of CCA cells as compared with control cells at 12 and 18 h (Figure 4B), which was confirmed by the quantification of the percentage of migration area (Figure 4C, *P* < 0.05). These results suggest that the overexpression of EBF1 could reduce the migration ability of CCA cells.

The effect of EBF1 overexpression on the proliferation of CCA cells was evaluated using SRB assay. As shown in Figure 4D, cell proliferation was significantly decreased in KKU-213A cells transiently transfected with EBF1 vector as compared with control cells at 48 and 72 h. To investigate the role of EBF1 protein on the invasion ability of KKU-213A cells, we used transwells coated with extracellular matrix. The results indicated that the number of invaded cells that attached to the lower surface of the membrane was significantly lower in EBF1-transfected KKU-213A than that of the control cells (*P* < 0.05), as shown in Figure 4E and 4F.

### The identification of EBF1-related pathways in CCA, and IL-6-related gene expression in EBF1-overexpressing CCA cells

**Figure 5:**
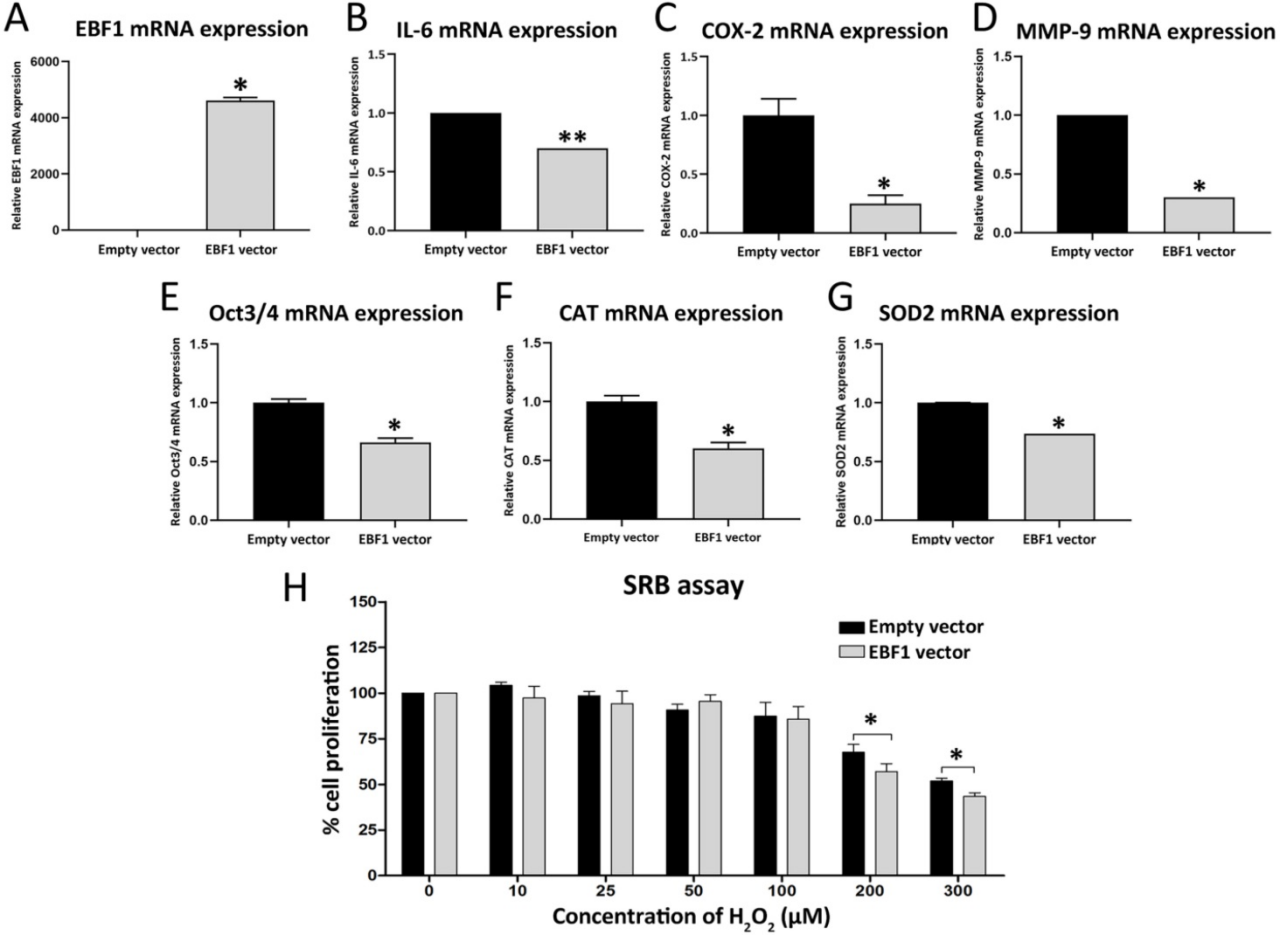
Relative mRNA expression levels of EBF1 (A), IL-6 (B), COX-2 (C), MMP-9 (D), Oct3/4 (E), CAT (F), SOD2 (G) adjusted by β-actin mRNA expression. (H) The graph represents cell densities of EBF1 overexpressing-KKU-213A cells (gray bar) compared with vehicle control (black bar) after exposure to various concentration of H_2_O_2_. *P* values are indicated by the asterisk, (*) *P* < 0.05 and (**) *P* < 0.001.

RNA sequencing analysis (RNA-seq) was performed from 2 sets of control KKU-213A cells and EBF1-overexprssing KKU-213A cells. Lists of the Entrez gene ID (747 of down-regulated genes and 552 of up-regulated genes) including genes that are significantly changed (*P* < 0.05 analyzed by Student's t-test, Supplementary Table S3) were subjected to DAVID gene annotation (https://david.ncifcrf.gov) for conducting the pathway enrichment analyses (shown in Table S6 and S7). The Kyoto Encyclopedia of Genes and Genomes (KEGG) analysis demonstrated that EBF1 suppressed “Pathway in Cancer”. In the pathway, EBF1 suppressed interleukin 6 (IL-6, *P* = 0.018), inducible nitric oxide synthase (iNOS or NOS2, *P* = 0.034) and epidermal growth factor (EGF, *P* = 0.042). We further confirmed the gene expressions by RT-qPCR, and found that IL-6 (Figure 5B) was significantly down-regulated in EBF1-overexpressing KKU213A cells (Figure 5A), but NOS2 and EGF were not significant different (data not shown). Therefore, IL-6-related genes in CCA were further investigated. IL-6 was previously reported to induce the expressions of COX-2 34, 35, MMP-9 36, Oct3/4 37, SOD2 38, 39 and CAT 39 in cancer cells. Therefore, we further investigated the expression levels of COX-2, MMP-9, Oct3/4, SOD2 and CAT in the EBF1-overexpressed CCA cells by RT-qPCR. We found that COX-2 (Figure 5C), MMP-9 (Figure 5D), Oct3/4 (Figure 5E), CAT (Figure 5F), SOD2 (Figure 5G) were significantly decreased in EBF1-overexpressing KKU-213A cells compared with their controls.

The expression of CAT and SOD2 are involved in antioxidant system in various cancer cells. In order to elucidate the roles of EBF1 expression in relation to oxidative stress-resistance properties of CCA cancer cells, EBF1-overexpressing KKU-213A cells were exposed to different concentrations of H_2_O_2_. Cell densities were significantly decreased in EBF1-overexpressing KKU-213A cells compared with control cells transfected with empty vector after exposure to high concentrations of H_2_O_2_ (200 and 300 μM) for 72 h (Figure 5H). These results suggest that EBF1 could decrease the oxidative stress-resistance property of CCA cells.

## Discussion

One of the hallmarks of cancer is the dysregulation of the DNA methylation machinery seeing as aberrant DNA methylation patterns 40. DNA methylome profiles of breast cancers revealed that EBF1 is an important transcription factor potentially involved in regulation of methylation states 41. In this study, DNA methylation level at the EBF1 promoter region was significantly higher in CCA tumors compared to NBD tissues using the DNA methylation array analysis. The methylation status of the promoter region of EBF1 gene using MSP analysis confirmed the results obtained from the DNA methylation array analysis. Moreover, MSP analysis revealed the significant correlation between high EBF1 promoter methylation and shorter survival rates of the CCA patients. The DNA methylation rates significantly higher in all CCA cell lines compared with the immortal cholangiocyte cell line, MMNK1. These data suggest that hypermethylation of EBF1 promoter region causes the down-regulation of EBF1 expression in CCA, leading to CCA progression with aggressive clinical outcomes.

The enzymes directly responsible for DNA methylation are DNMTs 42. Overexpression of DNMTs has been reported in several tumors and ultimately results in the hypermethylation of tumor suppressor genes 43. 5-Aza-dC is a well-known inhibitor of DNMTs 44. Several tumor suppressor genes are methylated in cancer cells and their expression can be induced with 5-Aza-dC treatment, leading to the reduction of the progression of tumor growth 45, 46. Kim and coworkers showed that EBF3, a tumor suppressor gene, was methylated in gastric carcinoma and its function could be restored with 5-Aza-dC treatment 47. DNMT inhibitors can reduce the progression of some CCA cell lines such as KKU-100, Mz-ChA-1, TFK-1 and QBC939 48-50. Our results showed that 5-Aza-dC treatment induced demethylation of EBF1 promoter region and increased EBF1 mRNA expression. Thus, the DNA methylation of EBF1 promoter by DNMT activation results in the suppression of the EBF1 expression. IL-6/STAT3 signaling pathway induces DNMT1 expression and is involved in epigenetic changes in CCA 51, 52. In our results, IL-6 was suppressed in EBF1-overexpressing CCA cells, suggesting that promoter hypermethylation of EBF1 in CCA may be activated by IL-6/STAT3 signaling pathway.

Suppression of EBF1 expression increased tumorigenic, stemness and oxidative stress-resistant properties of the cholangiocyte cells, suggesting that downregulation of EBF1 may be involved in the transformation of the cholangiocytes into CCA cells 12. In the present study, EBF1 expression in CCA cells was induced by the epigenetic therapeutic drug 5-Aza-dC, a DNMT inhibitor, treatment. Then, the functional roles of EBF1 were examined in CCA cells in order to confirm that restored EBF1 expression can suppress tumorigenic, stemness and oxidative stress-resistant properties in the cancer cells, suggesting possible application of epigenetic therapy for CCA treatment. Moreover, overexpression of EBF1 significantly reduced cell proliferation, migration and invasion as compared with the vehicle-treated control cells.

A pro-inflammatory cytokine, IL-6, and an oxidative stress response enzyme, cyclooxygenase 2 (COX-2), are involved in inflammatory processes and cancer progression 36, 51-57. Chronic inflammation induces not only NF-kB expression, but also its nuclear translocation, leading to activate IL-6 expression and Jak-STAT signaling pathway 35, 58, 59. IL-6 expression causes cancer progression via the activations of many IL-6 related pathway such as RAS and MAPK signaling 60, 61. Moreover, COX-2 can induce CCA progression through induction of MMP-9 whereas selective COX-2 inhibitors could inhibit the cancer cell progression 62-64. The overexpression of MMP-9 can promote cell proliferation through the downregulation of the expression of tumor suppressor genes in renal and glioblastoma cell lines 65, 66. Related to cell migration and invasion, epithelial-mesenchymal transition (EMT) is a complex process wherein the epithelial cells acquire the characteristics of invasive mesenchymal cells 67. MMP-9 is associated with the EMT process and is a well-known marker of EMT 67, 68. The overexpression of MMP-9 contributes to enhance the invasion and migration of CCA cells 69-71. Our results showed that IL-6, COX-2, MMP-9 as well as RAS and MAPK signaling pathways were significantly reduced in EBF1-overexpressing CCA cells. Taken together, EBF1 suppressed cell proliferation, migration and invasion via down-regulations of IL-6, COX-2 and MMP-9 along with their related pathways such as RAS and MAPK signaling pathways.

EBF1 had been found to induce stem cells differentiation. EBF1 triggers common lymphoid progenitor cells differentiation to pre-mature B cells (pro-B cells) and induced mesenchymal stem cell differentiation to adipocytes and osteocytes 9. Oct3/4 is expressed in embryonic stem cells, adult stem cells and tumor cells but not in the differentiated cells 72. Oct3/4 expression was induced by Wnt/β-catenin signaling pathway and IL-6/STAT3 signaling pathway 37, 73-75. Moreover, MMP-9, COX-2 and IL-6 can promote the induction of stem cell properties of cancer cells 37, 76, 77. Stem cells protect their genomic stability by maintaining low reactive oxygen species (ROS) via the up-regulation of antioxidant system 78. Superoxide dismutase 2 (SOD2) expressions is induced by Oct3/4 in the induced pluripotent stem (iPS) cells 79. SOD and catalase (CAT) are antioxidant genes regulated by Nrf2 transcription factor and are activated by reactive oxygen species (ROS) as well as oxidative stress 80, 81. In this study, EBF1 overexpression decreased IL-6 related pathways and the expressions of Oct3/4, SOD2 and CAT leading to loss of stemness and oxidative stress-resistant properties of CCA cells.

**Figure 6:**
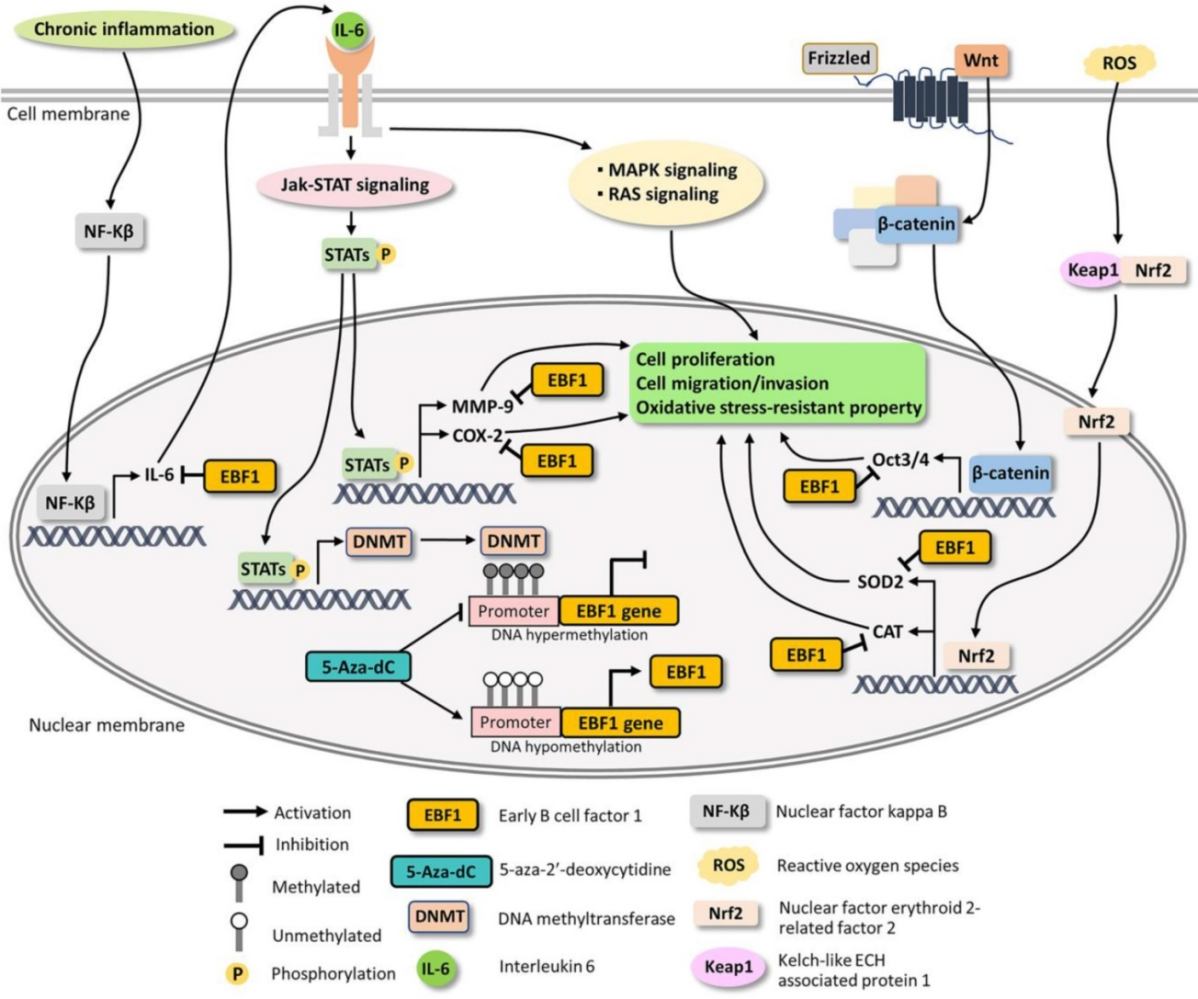
Possible mechanisms underlying EBF1 expression in CCA cells.

Based on our findings and literature reviews, the possible mechanisms underlying EBF1 expression in CCA are proposed in Figure 6. Our findings indicate that oxidative stress and promoter hypermethylation are synergistic causes of EBF1 suppression in CCA. EBF1 promoter hypermethylation may be regulated by IL-6/STAT3 signaling pathway. EBF1 inhibits IL-6 expression leading to the inhibitions of Jak-STAT, RAS and MAPK signaling pathways as well as COX-2 and MMP-9 expressions resulting in the inhibition of cancer progression. In addition, EBF1 exhibits tumor suppressive properties in CCA cells through decrease of stemness and oxidative stress-resistant properties via suppressions of Wnt signaling pathway and antioxidants via inhibition of Oct3/4, SOD2 and CAT expression. We therefore conclude that the epigenetic therapy could increase EBF1 expression, which contribute to reduce the tumor progression of the cancer cells.

## Supplementary Material

Supplementary figures and tables 1, 2, 4-7.

Supplementary table 3.

## Abbreviations

EBF1: Early B cell factor 1
CCA: cholangiocarcinoma
5-Aza-dC: 5-aza-2'-deoxycytidine
MSP: methylation-specific polymerase chain reaction
DNMT: DNA methyltransferase
NBD: normal bile ducts
