# Supplementary material for: Promoter hypermethylation of early B cell factor 1 (EBF1) is associated with cholangiocarcinoma progression
Source: J Cancer. 2021 Mar 5;12(9):2673–86. doi: 10.7150/jca.52378 (PMC8040704; doi:10.7150/jca.52378)
Supplement: Supplementary file 1 — Supplementary figures and tables 1, 2, 4-7. [file jcav12p2673s1.pdf]

Supplementary Figure S1.

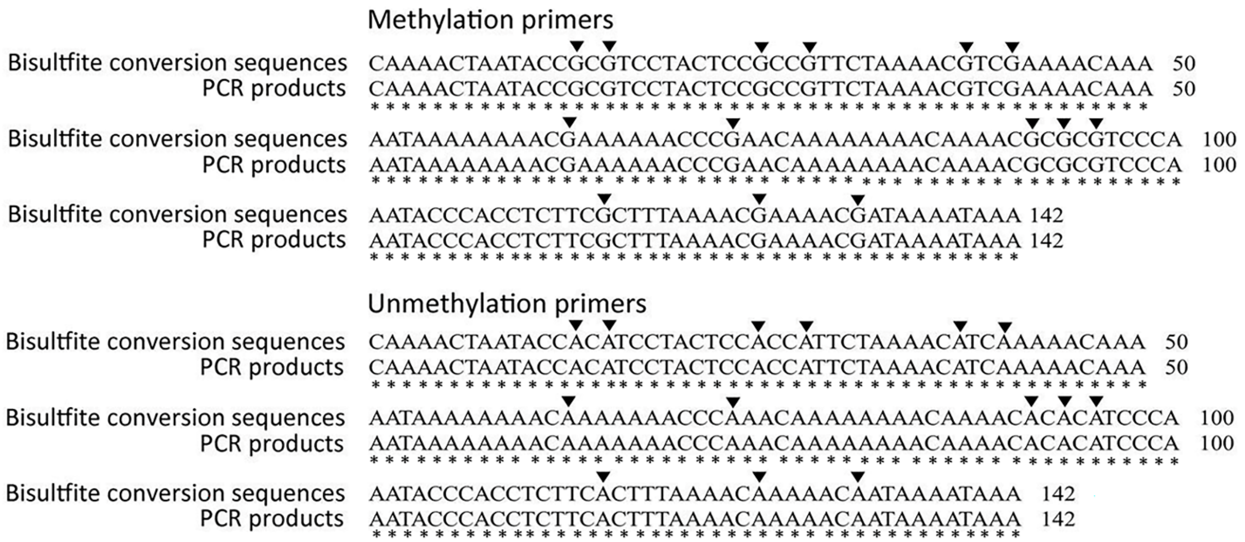

Figure S1 DNA sequencing results of PCR products from methylation and unmethylation primers. The PCR products from both primers were matched (100%) with the targeting bisulfite conversion sequences. Arrow heads indicate the CpG sites.

List of Supplementary Tables

Table S1. Primers for MSP of the *EBF1* gene

Type of primer	Forward primer (5' to 3')	Reverse primer (5' to 3')	Product size (bp)
**EBF1 M**	5'-TTT-ATT-TTA-TCG-TTT-TCG-TTT-TAA-AGC-G-3'	5'-CAA-AAC-TAA-TAC-CGC-GTC-CT-3'	142 bp
**EBF1 U**	5'-TTT-ATT-TTA-TTG-TTT-TTG-TTT-TAA-AGT-3'	5'-CAA-AAC-TAA-TAC-CAC-ATC-CT-3'	142 bp

Abbreviations: MSP: methylation-specific PCR, M: methylation primer, U: unmethylation primer

Table S2. MSP conditions of EBF1 gene

**Components#**	**Final concentration**	**PCR reaction**
$MgCl_2$	5.5 mM	Pre-heating: 95 °C 2 min
dNTP	0.2 mM	Denaturing: 95 °C 15s
PCR buffer	1x	Annealing:
Forward primer (met/unmet)	0.3 µM	-met primer 68 °C 30s ⎤
Reward primer (met/unmet)	0.3 µM	-unmet primer 60 °C 30s ⎬ 35 cycles
Taq DNA polymerase	1.5 U	Extension: 72 °C 30s ⎦
DNA sample	100 ng	Final extension: 72 °C 10 min

#$MgCl_2$ (Invitrogen, MA, USA), dNTP (Invitrogen, MA, USA), 10x PCR buffer (Invitrogen, MA, USA), and Taq DNA polymerase (Invitrogen, MA, USA)

Table S4. The correlations between DNA methylation status at EBF1 promoter region and EBF1 mRNA expression levels in CCA tissues (n=109) using Spearman's correlation coefficient

Correlations	EBF1 probes located at EBF1 promoter region										
	Transcription Start Site (TSS) 1500								First Exon		
	cg 01135 780	cg 05056 120	cg 00251 610	cg 15963 563	cg 05774 756	cg 16126 280	cg 16317 459	cg 26664 797	cg 0553 0568	cg 0421 7450	cg 1700 9297
**EBF1 mRNA expression levels**	-0.313*	-0.306*	-0.241*	-0.223*	-0.175	0.043	-0.129	0.006	-0.054	-0.112	-0.114

P values are indicated by the asterisk, (*) $P < 0.05$ by Spearman's correlation coefficient.

Table S5. Association of methylation status of EBF1 promoter region and clinicopathological data in CCA patients

**Clinical data**	**EBF1 methylation status**		
	**Low DNA methylation (n=18)**	**High DNA methylation (n=54)**	***P*-value**
Age			
< 57	12	28	0.206
≥ 57	6	26	
Sex			
Male	11	38	0.326
Female	7	16	
Histological			
Non-papillary type	11	33	0.606
Papillary type	7	21	
Metastasis status			
Non-metastasis	10	23	0.247
Metastasis	8	31	
Median survival (days)	374	243	0.023*

**P*-value < 0.05; analyzed by Pearson's Chi-square test and log-rank test

Table S6. The down-regulated pathways in EBF1-overexpressing CCA cells

**No.**	**KEGG pathway terms**	**Genes**	**Genes count**	***P*-value**
1	Ubiquitin mediated proteolysis	SMURF1, XIAP, ANAPC1, BIRC3, CUL2, CUL3, KLHL9, TRIP12, UBE2K, UBE2Q2, UBE2Z, UBE3A, UBE3C	13	1.8E-3
2	Pathways in cancer	CTBP2, CRK, E2F3, GNA12, GNG11, RALB, ROCK2, SMAD2, SOS2, WNT2B, XIAP, BIRC3, CUL2, CCND1, EGF, FGF19, FGFR2, FZD8, IL6, MAPK1, MAPK8, NRAS, NOS2, PPARD, STAT1	25	2.5E-3
3	Pancreatic cancer	E2F3, RALB, SMAD2, CCND1, EGF, MAPK1, MAPK8, STAT1	8	5.3E-3
4	Choline metabolism in cancer	PDPK1, SOS2, DGKE, EGF, GPCPD1, MAPK1, MAPK8, NRAS, PLA2G4E, SLC22A3	10	5.9E-3
5	Chronic myeloid leukemia	CTBP2, CRK, E2F3, SHC4, SOS2, CCND1, MAPK1, NRAS	8	9.3E-3
6	Non-small cell lung cancer	PDPK1, E2F3, SOS2, CCND1, EGF, MAPK1, NRAS	7	9.9E-3
7	Bladder cancer	E2F3, CCND1, EGF, MAPK1, NRAS, RPS6KA5	6	1.1E-2
8	FoxO signaling pathway	PDPK1, BCL2L11, SMAD2, SOS2, CCND1, EGF, IL6, MAPK1, MAPK8, NRAS, PRKAB2	11	1.3E-2
9	Tuberculosis	ATP6V0D2, BCL10, ITGAM, IL6, HLA-DPA1, MAPK1, MAPK8, NOS2, NFYB, PPP3R1, RIPK2, STAT1, TLR4	13	1.4E-2
10	Glioma	E2F3, SHC4, SOS2, CCND1, EGF, MAPK1, NRAS	7	2.0E-2
11	MAPK signaling pathway	CRK, GNA12, RASGRF2, SOS2, DUSP8, EGF, FGF19, FGFR2, MAPK1, MAPK8, MAPKAPK5, MRAS, NRAS, PLA2G4E, PPP3R1, RPS6KA5	16	2.0E-2
12	Hepatitis B	E2F3, CCND1, IFNAR1, IFIH1, IL6, MAPK1, MAPK8, NRAS, STAT1, TLR3, TLR4	11	2.1E-2
13	Prostate cancer	PDPK1, E2F3, SOS2, CCND1, EGF, FGFR2, MAPK1, NRAS	8	2.6E-2
14	Endometrial cancer	PDPK1, SOS2, CCND1, EGF, MAPK1, NRAS	6	2.8E-2
15	Toxoplasmosis	PDPK1, XIAP, BIRC3, HLA-DPA1, MAPK1, MAPK8, NOS2, STAT1, TLR4	9	2.9E-2
16	Influenza A	OAS1, ADAR, IFNAR1, IFIH1, IL6, HLA-DPA1, MAPK1, MAPK8, NUP98, STAT1, TLR3, TLR4	12	2.9E-2

17	Prolactin signaling pathway	SHC4, SOS2, CCND1, MAPK1, MAPK8, NRAS, STAT1	7	2.9E-2
18	Leishmaniasis	ITGAM, HLA-DPA1, MAPK1, NCF2, NOS2, STAT1, TLR4	7	2.9E-2
19	Hepatitis C	OAS1, PDPK1, SOS2, EGF, IFNAR1, MAPK1, MAPK8, NRAS, STAT1, TLR3	10	3.1E-2
20	Measles	OAS1, CD28, ADAR, CSNK2A1, CCND1, IFNAR1, IFIH1, IL6, STAT1, TLR4	10	3.1E-2
21	Ras signaling pathway	GNG11, RALB, RASGRF2, SHC4, SOS2, EGF, FGF19, FGFR2, MAPK1, MAPK8, MRAS, NRAS, PLA2G4E, PLCE1	14	3.6E-2
22	Wnt signaling pathway	CTBP2, ROCK2, WNT2B, CSNK2A1, CCND1, FZD8, MAPK8, PPARD, PPP3R1, SOST	10	3.8E-2
23	Focal adhesion	PDPK1, CRK, ROCK2, SHC4, SOS2, XIAP, BIRC3, CCND1, EGF, ITGA10, ITGA7, MAPK1, MAPK8	13	4.0E-2
24	Signaling pathways regulating pluripotency of stem cells	SMAD2, WNT2B, BMPR2, FGFR2, FZD8, KAT6A, MAPK1, NRAS, PCGF5, PCGF6	10	4.2E-2
25	Neurotrophin signaling pathway	PDPK1, CRK, SHC4, SOS2, MAPK1, MAPK8, NRAS, RIPK2, RPS6KA5	9	4.4E-2
26	Regulation of actin cytoskeleton	CRK, GNA12, ROCK2, SOS2, EGF, FGF19, FGFR2, ITGA10, ITGA7, ITGAM, MAPK1, MRAS, NRAS	13	4.4E-2

Table S7. The up-regulated pathways in EBF1-overexpressing CCA cells

No.	KEGG pathway terms	Genes	Genes count	*P*-value
1	Metabolic pathways	OXSM, PGLS, ATP5G1, ATP6AP1, CDIPT, COASY, DAO, NADSYN1, NSDHL, NDUFV1, NDUFA9, NDUFB7, NME4, NME6, POLR2J, ADK, AMPD2, AKR1A1, AKR1C3, AMACR, ACY1, AMY1C, B4GALT2, BST1, COX4I1, CYC1, FAH, G6PD, GLS2, GSTZ1, GRHPR, HAL, HSD17B2, KMO, LIPT2, MDH2, MAN1B1, MECR, NAPRT, PEMT, PIGY, PLPP2, PAFAH1B3, PRODH, SDS, TST, UQCRC1, UROD	48	6.8E-4
2	Huntington's disease	ATP5G1, NDUFV1, NDUFA9, NDUFB7, POLR2J, TBP, COX4I1, COX7A2L, CYC1, IFT57, SLC25A6, UQCRC1	12	7.5E-3
3	Biosynthesis of antibiotics	PGLS, DAO, NSDHL, NME4, NME6, AMPD2, AKR1A1, ACY1, G6PD, MDH2, PRODH, SDS	12	1.5E-2
4	Oxidative phosphorylation	ATP5G1, ATP6AP1, NDUFV1, NDUFA9, NDUFB7, COX4I1, COX7A2L, CYC1, UQCRC1	9	1.6E-2
5	Parkinson's disease	ATP5G1, NDUFV1, NDUFA9, NDUFB7, COX4I1, COX7A2L, CYC1, SLC25A6, UQCRC1	9	2.3E-2
6	Systemic lupus erythematosus	H2AFZ, C3, HIST1H4H, HIST2H3D, HLA-DMA, HLA-DRA, SNRPD3, SNRPB	8	4.7E-2
